# Compression-Induced Tensile Mechanical Behaviors of the Crystalline Rock under Dynamic Loads

**DOI:** 10.3390/ma13225107

**Published:** 2020-11-12

**Authors:** Bowen Zheng, Shengwen Qi, Xiaolin Huang, Ning Liang, Songfeng Guo

**Affiliations:** 1 Key Laboratory of Shale Gas and Geoengineering, Institute of Geology and Geophysics, Chinese Academy of Sciences, Beijing 100029, China; zhengbowen@mail.iggcas.ac.cn (B.Z.); huangxiaolin@mail.iggcas.ac.cn (X.H.); liangning@mail.iggcas.ac.cn (N.L.); guosongfeng@mail.iggcas.ac.cn (S.G.); 2 Innovation Academy for Earth Science, Chinese Academy of Sciences, Beijing 100029, China; 3 College of Earth and Planetary Sciences, University of Chinese Academy of Sciences, Beijing 100049, China

**Keywords:** crystalline rock, compression-induced tensile behavior, dynamic load, microstructure, grain heterogeneity, grain-based model, UDEC

## Abstract

Characterization of the tensile mechanical behaviors of rocks under dynamic loads is of great significance for the practical engineering. However, thus far, its micromechanics have rarely been studied. This paper micromechanically investigated the compression-induced tensile mechanical behaviors of the crystalline rock using the grain-based model (GBM) by universal distinct element code (UDEC). Results showed that the crystalline rock has the rate- and heterogeneity-dependency of tensile behaviors. Essentially, dynamic Brazilian tensile strength increased in a linear manner as the loading rate increased. With the size distribution and morphology of grain-scale heterogeneity weakened, it increased, and this trend was obviously enhanced as the loading rate increased. Additionally, the rate-dependent characteristic became strong with the grain heterogeneity weakened. The grain heterogeneity prominently affected the stress distribution inside the synthetic crystalline rock, especially in the mixed compression and tension zone. Due to heterogeneity, there were tensile stress concentrations (TSCs) in the sample which could favor microcracking and strength weakening of the sample. As the grain heterogeneity weakened or the loading rate increased, the magnitude of the TSC had a decreasing trend and there was a transition from the sharp TSC to the smooth tensile stress distribution zone. The progressive failure of the crystalline rock was notably influenced by the loading rate, which mainly represented the formation of the crushing zone adjacent to two loading points. Our results are meaningful for the practical engineering such as underground protection works from stress waves.

## 1. Introduction

To date, the drilling and blasting method is the most commercial and efficient way to excavate the rock mass in the practical engineering, such as tunneling and mining [1,2]. Blasting can induce the dynamic tensile stress, which splits the rock mass and migrates fragments toward the free face. On the other hand, the blasting or strong seismic compressive stress waves can be converted into tensile stress waves [3,4] when reflecting from the underground chamber wall or the slope surface. These tensile stress waves can potentially make the engineering rock mass produce tensile failure. It is thus of great significance to examine the dynamic tensile behaviors of rocks for the design of the rock engineering and the evaluation of the rock mass stability.

Numerous laboratory tests were conducted to investigate the tensile behaviors of rocks under quasi-static loads. Initially, the uniaxial tension test is a direct method to explore the tensile behaviors of the rock materials [5,6]. However, it is often difficult or inconvenient to process a dog-bone tensile sample and to tightly fix the sample end with the loading cell. A common substitute for the direct tension test is the Brazilian test [7], which was proposed primarily to avoid the need to carry out direct tension tests. As an indirect tension test, the Brazilian tensile test is conducted by the compression-induced tensile stress along the central line of the disc sample by applying linear compressive loads to the contacts between the sample and the loading cell. Today, the Brazilian test is the suggested standard method to measure rock tensile strength by the International Society of Rock Mechanics (ISRM) [8]. Due to the convenience in both the sample preparation and the loading process, a series of Brazilian tests were conducted to characterize the tensile behaviors of rocks in previous studies. Results showed that the tensile behaviors of rocks highly depended on the mineral phase and grain-scale structures [9,10,11]. Especially for the crystalline rock, it is often made up of different types of mineral grains which possess inherent heterogeneous microstructures. These microstructures are associated with mineral aggregations and microdefects such as cleavage planes, voids, and micro cracks [12,13,14], which possess geometric, physical, mechanical, and locational grain-scale heterogeneity. These heterogeneous microstructures have an important effect on the macro-mechanical characteristics of the crystalline rock such as the deformation, strength, and cracking behavior [9,10,15,16,17,18,19,20,21,22]. It is also worth noting that the heterogeneity inside the crystalline rock resulted in tensile stresses and concentration zones, even if the rock was loaded by the purely compressive stress [23,24]. Meanwhile, some grain-based models (GBM) were adopted to numerically capture the effect of heterogeneity on compression-induced tensile behaviors of the crystalline rock under quasi-static loads [9,10,11].

Out of the quasi-static case, the Brazilian test was extended to the dynamic case by making full use of the split Hopkinson pressure bar (SHPB) system [25,26,27]. Besides the internal heterogeneous microstructure, the loading rate significantly influenced the tensile behaviors of the rock materials. It was found that the Brazilian tensile strength increased with the loading rate increasing [26]. Near two loading points on the sample, there were two obvious crushing zones with a “V” shape, of which the areas increased as the loading rate increased. Additionally, the number (or size) of fragments increased (decreased) [27]. Reference [28] studied the compression-induced tensile behaviors of pre-crack Brazilian disc samples and found that both the loading rate and the inside fracture controlled the tensile mechanical behaviors of the sample. Reference [29] investigated the progressive failure process of disc Carrara marble samples under the dynamic compression-induced tensile stress. However, thus far, very few studies have reported on the influence of grain-scale heterogeneity on the dynamic tensile characteristics of the crystalline rock, such as the compression-induced tensile stress distribution, the tensile strength, and the cracking behavior from the micromechanical viewpoint. The objective of this study was to investigate the effect of grain heterogeneity on the dynamic compression-induced tensile behaviors of the crystalline rock, combining SHPB tests and fine numerical simulations based on GBM implemented by the universal distinct element code (UDEC) [30,31]. Compared with the contact dynamics (CD) and force-based molecular dynamics (FBMD) simulation methods to study granular materials [32,33], UDEC can generate the deformable mineral grain and consider the heterogeneity of the grain morphology directly. During this research, the grain heterogeneity was mainly incorporated by varying the size distribution and morphology of the minerals in GBM.

This paper was structured as follows. Section 1 reviewed previous studies on tensile behaviors of the brittle rock and analyzed the limitations in these studies. Section 2 introduced the experimental methods and numerical models on the dynamic compression-induced tensile tests of the crystalline rock. Section 3 showed the results in detail and these results were discussed in Section 4. Conclusions were given in Section 5.

## 2. Materials and Methods

### 2.1. Sample Preparation and Experimental Procedure

We adopted granite as the testing material in this study, which was collected from the Shuangjiangkou (SJK) hydropower station in the Aba prefecture, Sichuan Province, Southwest China. The collected granite rock cores were processed as disc samples to conduct dynamic Brazilian tensile tests with a diameter of 50 mm and a thickness of 25 mm. Firstly, the rock samples were drilled along the radial direction of the granite rock cores (about diameter of 150 mm) to form cylinders with a nominal diameter of 50 mm. Then, the rock cylinders were cut from their radial direction into disc samples with a nominal thickness of 25 mm. Subsequently, the disc samples were polished for their top, bottom, and side surfaces by the grinding machine, which insured the surface undulation of less than 0.1 mm (about 0.4% of the thickness). The SJK granite mainly has a grain size ranging from 1.1 to 6.9 mm (covering more than 95% of grains) and has a mean value of 1.8 mm. It has a density of 2620 kg/m^3^, Young’s modulus of 60 GPa, and Poisson’s ratio of 0.1. Fractures were invisible on the sample surface to the naked eye. The mineral composition analysis results showed that it primarily consisted of 40% K-feldspar, 25% quartz, 30% plagioclase, and 5% biotite.

**Figure 1:**
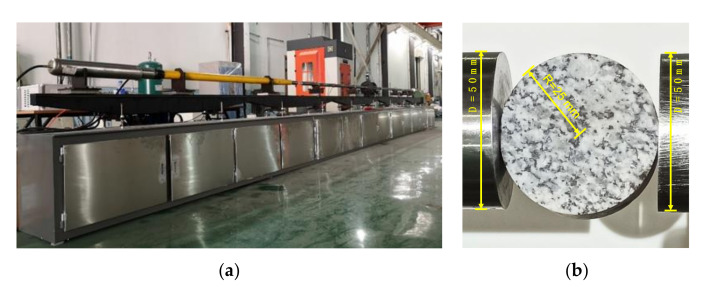
(**a**) The split Hopkinson pressure bar (SHPB) testing system; (**b**) a Brazilian disc sample sandwiched between the incident bar and the transmitted bar.

The dynamic Brazilian tensile tests were carried out by the steel SHPB system (Figure 1a), which is located in the Key Laboratory of Shale Gas and Geoengineering, Institute of Geology and Geophysics, Chinese Academy of Sciences. The SHPB system mainly consists of a gas gun, a fusiform striker, an incident bar, a transmitted bar, and an absorbing bar. Both the incident and transmitted bar have a diameter of 50 mm and a length of 2000 mm. The disc sample was sandwiched between the incident bar and the transmitted bar, as shown in Figure 1b. High-pressure air was initially stored in the gas gun. When the gas gun was triggered, the high-pressure gas was suddenly released and it accelerated the fusiform striker. The striker flew forward with a certain velocity and then impacted the incident bar end, producing a compressive stress pulse. A larger gas pressure could induce a larger amplitude stress pulse. Five groups of samples were loaded by the respective gas pressure of 0.24, 0.26, 0.28, 0.30, and 0.32 MPa.

The induced stress wave first propagated in the incident bar (incident wave). When impinging on the sample, one portion of the stress wave traveled into the transmitted bar (transmitted wave) while the other portion was reflected (reflected wave). The sample was loaded to fail under these three stress waves. The incident and transmitted waves were recorded by one pair of strain gages placed in the middle of the incident bar. The transmitted wave was recorded by the other pair located in the point with 1500 mm length from the end of the transmitted bar. Each pair of strain gages were mounted symmetrically on the bar surface across the bar diameter. The signals from each pair of strain gages were conditioned with a Wheatstone bridge to better the accuracy of the testing data. Data were recorded at 10 MHz and 12 bits digital resolution.

On the condition that the forces applied to the front and rear contacts of the sample keep balance, the dynamic split tensile stress *σ_s_*(*t*) in the vertical central plane of the sample can be calculated according to the following formula [8].

(1) $$\sigma_{s}\left(t\right)=\frac{2F\left(t\right)}{\pi Dh},$$

where, *F*(*t*) denotes the linear force applied to the sample; *D* is the diameter of the sample; *h* is the thickness of the sample; *t* stands for the time.

Because the transmitted stress wave *σ_T_*(*t*) is the actual load subjected to the sample, we can determine *F*(*t*) according to the following equation.

(2) $$F\left(t\right)=\frac{1}{4}\pi D^{2}\sigma_{T}\left(t\right).$$

The peak of *σ_s_*(*t*) is defined as the dynamic tensile strength of the rock. The slope of the linear portion of *σ_s_*(*t*) in the loading stage is defined as the loading rate. Some details are shown in the following figure.

### 2.2. Numerical Modelling

**Figure 2:**
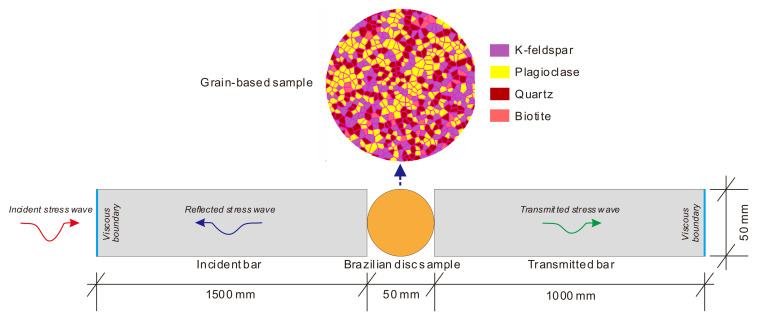
Configuration of the universal distinct element code (UDEC) model corresponding to the dynamic Brazilian tensile test by SHPB. For better presentation, the bar length does not match with the actual one. For interpretation of the references to color in this figure, the reader is referred to the electronic version of this paper.

Since the experimental method was not enough to micromechanically investigate the dynamic compression-induced tensile behaviors of the crystalline rock, we combined the numerical method based on GBM to solve the problem in this study. The numerical simulations were implemented by UDEC 6.0, which is a commercial software developed by the Itasca Consulting Group Inc. [31]. The UDEC is a numerical program based on the distinct element method for discontinuum modeling. UDEC uses an explicit time-marching scheme to solve the equations of motion directly [30,31]. Considering the sample dimension, mineral compositions, and mean grain size of the SJK granite, we first built a 2D synthetic GBM sample, as shown in Figure 2, based on the Voronoi tessellation technique in UDEC [31].

The GBM sample in UDEC was made up of polygons with a mean grain size of 1.91 mm (the major axis length of the polygon). Each polygonal grain possessed its identity, which could represent one mineral. The four mineral compositions followed the uniformly random distribution in GBM, the types and contents of which were consistent with those of the mineral constituents in the actual rock. Table 1 shows the comparison of the mineral compositions between the actual rock and the synthetic rock by GBM. The polygonal grains were deformable and their insides were discretized by utilizing triangular meshes. The grains were bonded with each other along their contact interfaces.

The calculations of the GBM sample in UDEC alternated between the application of a force-displacement law at all contact interfaces and Newton’s second law at all polygonal grains [30,31]. The force-displacement law was used to find contact forces from known displacements. Newton’s second law gave the motion of the grains resulting from the known forces acting on them, i.e., the motion was calculated at the gridpoints of the triangular finite-strain elements within the grains.

Each polygonal grain was treated as a purely elastic material which could not fail regardless of the external loads. Elastic properties of different minerals were taken according to Table 2. Contact interfaces among grains were breakable when the tensile or shear stress exceeded the tensile or shear strength of the grain contact, which was depicted by the Mohr–Coulomb slip model. The involved parameters were tensile strength (*T*), cohesion (*c*), and friction angle (*θ*). The grain contact could only produce the linear deformation before failure, which was controlled by the normal (*k*_n_) and shear (*k*_s_) stiffness. After failure, the grain contact could open and slip along the interface.

Note that the bar length in the numerical model was shorter than that of the actual SHPB. This is because the practical SHPB should adopt the incident and transmitted bar with the enough length to avoid interference from the reflected wave, while the reflected wave could be absorbed by the viscous boundary in the numerical model. As shown in Figure 2, the left side of the incident bar and the right side of the transmitted bar were set to the viscous boundary, while other sides of the bars were set to the free boundary. The compressive stress pulse was input at the left boundary of the numerical model as the incident stress wave. The 2D model has a unit thickness in UDEC. Thus, Equation (2) has to be changed into the following equation.

(3) $$F\left(t\right)=wh\sigma_{T}\left(t\right),$$

where, *w* is the width of the numerical model that is taken as 50 mm; *h* is taken as the unit thickness of 1.0.

It is difficult to directly obtain the micro grain contact parameters involved in GBM by laboratory tests. Additionally, these micro-scale parameters are not directly related to the macro behaviors of the synthetic crystalline rock. Thus, micro-scale parameters in GBM need to be calibrated by trial and error until the numerical results agree well with the experimental ones. In this study, we first input the experimental stress pulse to simulate the actual case. Micro grain contact parameters were calibrated by comparing the tensile stress calculated from the experimental and simulated stress histories as well as the variation of the dynamic tensile strength versus the loading rate. To accurately control the incident stress pulse *σ*_I_(*t*) and better the force balance, we then adopted the half-sinusoidal stress wave as follows:

(4) $$\sigma_{I}\left(t\right)=A\sin\left(2\pi ft\right)\;\;(0<t<1/\left(2f\right)),$$

where, *A* denotes the amplitude of the stress wave; *f* is the frequency. By adapting *A* or *f*, we can achieve different loading rates applied to the sample.

## 3. Results

### 3.1. Experimental Results and the Numerical Model Calibration

**Figure 3:**
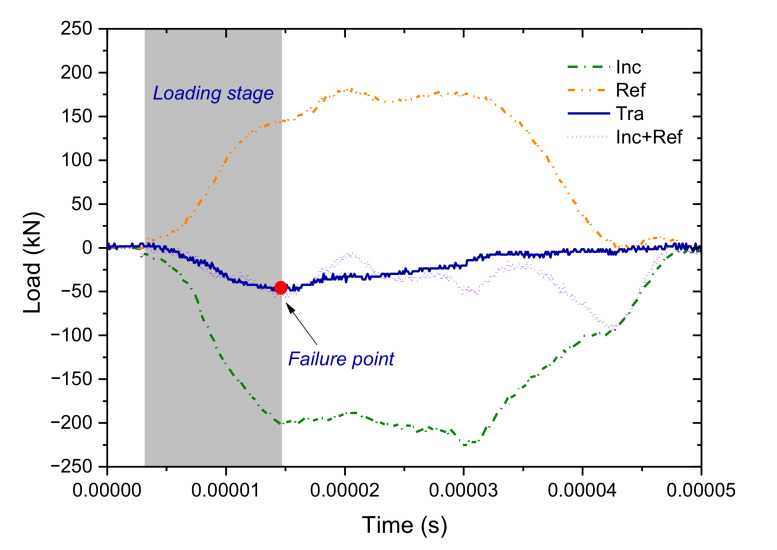
The force balance check at the front and rear contact interfaces of the disc sample. The incident stress wave was induced by the gas gun pressure of 0.24 MPa. For interpretation of the references to color in this figure, the reader is referred to the electronic version of this paper.

The balance condition of the force applied to the sample is fundamental to calculate the Brazilian tensile stress and strength. Because the wave propagation velocity was about 5000 m/s in the rocks, it took about 1 × 10^−5^ s for the wave propagation through the disc sample with a diameter of 50 mm. This indicated that the reflection and transmission of the stress wave approximately occurred synchronously. The incident, reflected, and transmitted waves were used to check the balance condition of the force applied to the sample, as shown in Figure 3. It can be seen that the summed force between the incident one and the reflected one essentially overlaps with the transmitted one in the loading stage. After the failure point, the summed force gradually deviates from the transmitted one. This indicates that the forces at the front and rear contacts of the sample essentially keep balance until the sample produces macro split failure by the tensile stress. In this paper, we mainly concerned the tensile stress distribution before the failure and the peak tensile strength. Hence, the stress balance condition fulfilled the requirement.

**Figure 4:**
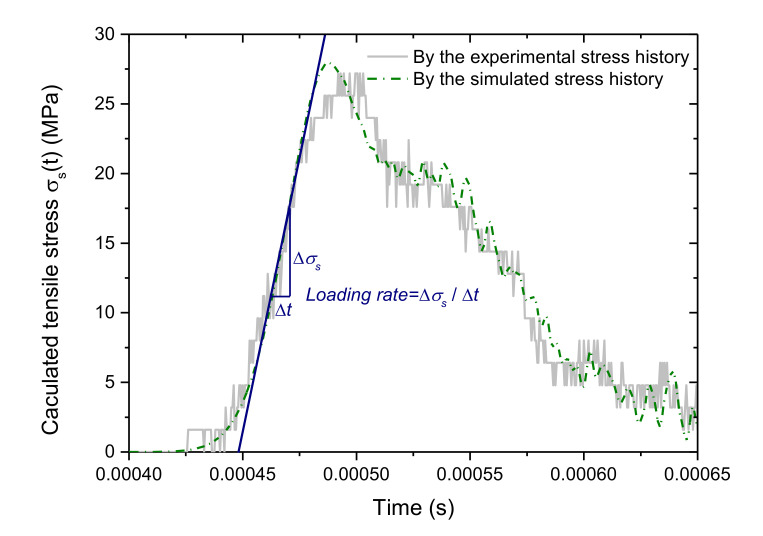
Comparison between the calculated tensile stress by the experimental stress history (gas pressure is 0.24 MPa) and by the simulated one. For interpretation of the references to color in this figure, the reader is referred to the electronic version of this paper.

After many trials, we acquired the calibrated parameters listed in Table 3, which could well reproduce the experimental results. Figure 4 shows the calculated tensile stress histories by the experimental transmitted stress wave and the simulated one, respectively. The tensile stress initially nonlinearly increases with time and it then essentially presents a linear manner. When it approaches the peak, the slope of the curve gradually decreases, which indicates the progressive failure processes of the sample. The simulated tensile stress history agrees well with the experimental one. This figure also clearly shows the definition of the loading rate as mentioned in Section 2.1.

**Figure 5:**
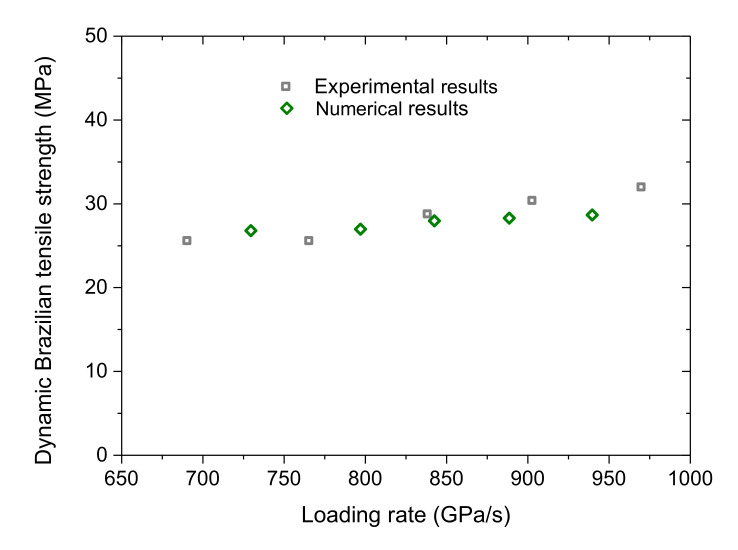
Comparison between the dynamic Brazilian tensile strength versus the loading rate by the experiment and by the numerical simulation. For interpretation of the references to color in this figure, the reader is referred to the electronic version of this paper.

Figure 5 shows the variation of the dynamic Brazilian tensile strength of the SJK granite versus the loading rate as samples suffered from the dynamic loads induced by the gas pressure of 0.24, 0.26, 0.28, 0.30, and 0.32 MPa, respectively. It can be seen that the dynamic Brazilian tensile strength increases with the loading rate essentially in a linear manner. The numerical results match well with the experimental data.

From Figure 4 and Figure 5, we can verify that the calibrated parameters are feasible to conduct numerical simulations on the dynamic compression-induced tensile mechanical behaviors of the crystalline rock. Note that the experimental stress waves in Figure 3 have some distortions on some portions, which affect the force balance of the sample. Reference [35] found that the approximately half-sinusoidal incident stress wave by the pulse shaper could promote the stress balance between two ends of the sample. Moreover, the half-sinusoidal wave can be readily achieved and controlled in the amplitude and duration in the numerical model. For these reasons, we adopted the half-sinusoidal wave in Equation (4) as the input stress pulse throughout the numerical simulations of the following sections.

### 3.2. Numerical Results

**Figure 6:**
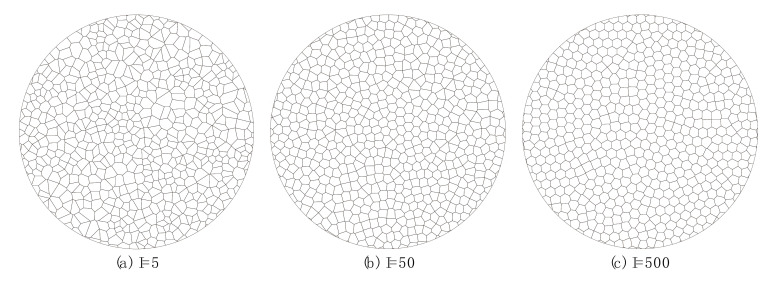
Synthetic disc crystalline rock samples with different grain heterogeneity. The average grain size for each sample is similar, i.e., (**a**) 1.91 mm; (**b**) 1.95 mm; (**c**) 2.00 mm. The mineral contents of three synthetic samples are the same as those of the SJK granite.

According to the calibrated parameters of GBM in Section 3.1, we further investigated the compression-induced tensile mechanical behaviors of the crystalline rock considering the loading rate and the heterogeneity of the grain morphology. According to the Voronoi tessellation generation technique in UDEC [31], the iteration number mainly controls the size distribution and morphology of grain-scale heterogeneity when the random seed and the average edge length of the polygon are fixed. The size distribution and morphology of Voronoi grains can be made more uniform by increasing the iteration number. In this study, we considered three types of synthetic samples considering different grain heterogeneity, as shown in Figure 6. The values of I (short for iteration numbers) of the three types of samples were 5, 50, and 500, respectively. Correspondingly, the total numbers of the mineral grains in the three types of samples were 644, 637, and 597, respectively. The time step during the simulation was 1.344 × 10^−9^ s, and the total running time of a model was about two and a half hours.

**Figure 7:**
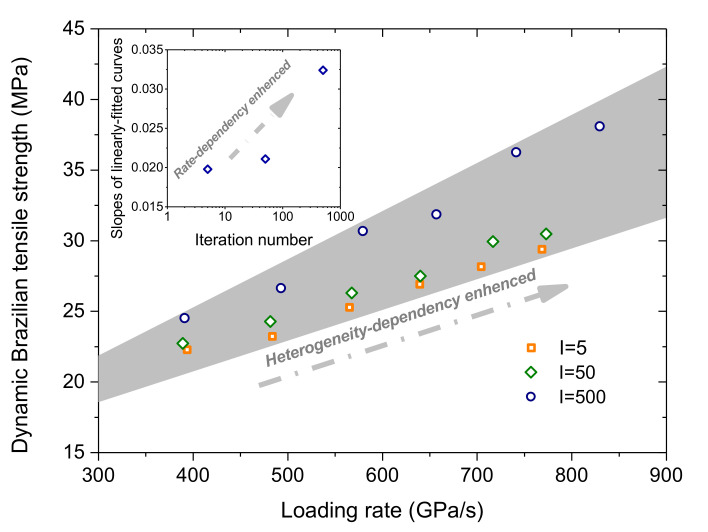
Variation of the dynamic Brazilian tensile strength versus the loading rate considering different grain heterogeneity. The subfigure corresponds to the variation of the slope of the linearly fitted curve with respect to the iteration number. A larger slope stands for a stronger rate-dependency degree. For interpretation of the references to color in this figure, the reader is referred to the electronic version of this paper.

According to Equation (4), we took a fixed frequency of 250 kHz but varied *A* to achieve different loading rates subjected to the sample. i.e., 80, 100, 120, 140, 160, and 180 MPa, respectively. Figure 7 shows that the dynamic Brazilian tensile strength of the sample depends on both the loading rate and its grain heterogeneity. The gray zone was enclosed by the upper and lower envelops of the data scope. With increasing the loading rate, the dynamic Brazilian tensile strength increases essentially in a linear manner. From the subfigure, it can be inferred that the rate-dependency of the Brazilian tensile strength is enhanced as the iteration number increases (the grain heterogeneity weakened). Under a similar loading rate, the dynamic Brazilian tensile strength increases with the grain heterogeneity weakened. When the iteration number ranges from 5 to 50 (coarse to good), the dynamic Brazilian tensile strength only has a relatively small increment. As it ranges from 50 to 500 (good to fine), the dynamic Brazilian tensile strength has a notably increasing trend. We can also observe an interesting phenomenon, that is, the gray zone gradually widens as the loading rate increases, which indicates that the heterogeneity-dependency of the dynamic Brazilian tensile strength was enhanced.

**Figure 8:**
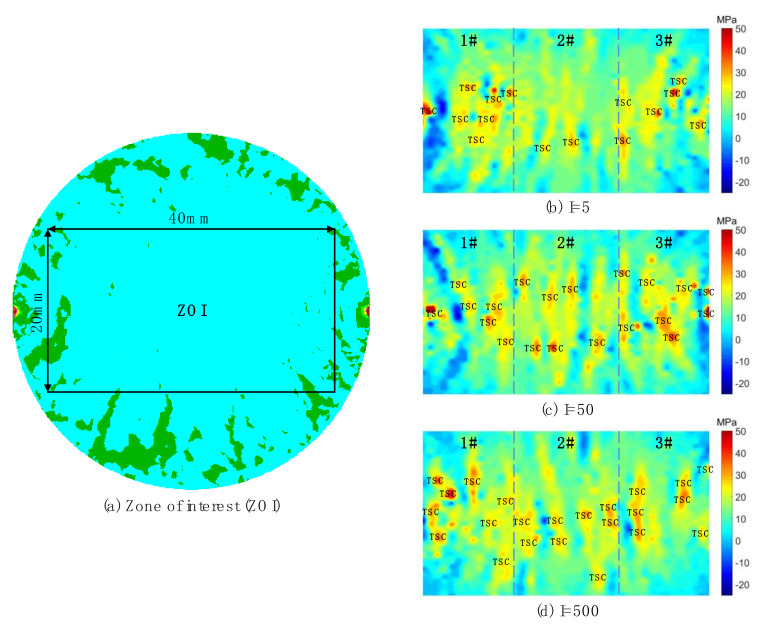
Distributions of the minor principal stress inside samples with different grain heterogeneity when bearing the displacement of 0.1 mm between two loading points. (**a**) Zone of interest; (**b**) I = 5 and the loading rate = 394 GPa/s; (**c**) I = 50 and the loading rate = 389 GPa/s; (**d**) I = 500 and the loading rate = 391 GPa/s. The positive presents the tensile stress and negative denotes the compressive stress. For interpretation of the references to color in this figure, the reader is referred to the electronic version of this paper.

To understand the influence of the grain heterogeneity on the compression-induced tensile behaviors of the crystalline rock, the stress distributions inside the synthetic sample were systematically analyzed. Figure 8 shows the distributions of the minor principal stress inside samples with different grain heterogeneity when the loading rate is around 390 GPa/s and the displacement between two loading points is 0.1 mm. Due to the large compressive stress adjacent to the loading point, as shown in Figure 8a, the stress distribution in other areas cannot be clearly displayed. For the Brazilian tension test, the compression-induced tensile stress mainly distributes along a narrow central zone of the sample, of which two margins are slightly away from two loading points. We thus enclosed a rectangle area as the zone of interest (ZOI) to better exhibit the stress distribution characteristics. From Figure 8b–d, it can be seen that each stress distribution is heterogeneous, which may result from the heterogeneity of both the grain morphology and mechanical properties. That is, there exists concentration zones of both the compressive and tensile stress in turn, which sharply vary in the stress field. When the grain heterogeneity is strong (I = 5), the tensile stress concentration (TSC) mainly distributes in subzones 1# and 3#. In subzone 2#, the tensile stress has a relatively homogenous distribution which is lack of the TSC, as shown in Figure 8b. It can be inferred that the tensile failure is more prone to initiate in subzones 1# and 3# in this case. As the grain heterogeneity weakens (I = 50) (Figure 8c), there are more TSCs appearing in subzone 2#. With the grain heterogeneity further weakened (I = 500) (Figure 8d), the TSC distributes in three subzones essentially in an equal manner. Compared with the cases in Figure 8b,c, there are less sharp TSCs in Figure 8d, which indicates that TSCs essentially have similar magnitudes. Thus, the tensile failure may initiate in three subzones synchronously.

**Figure 9:**
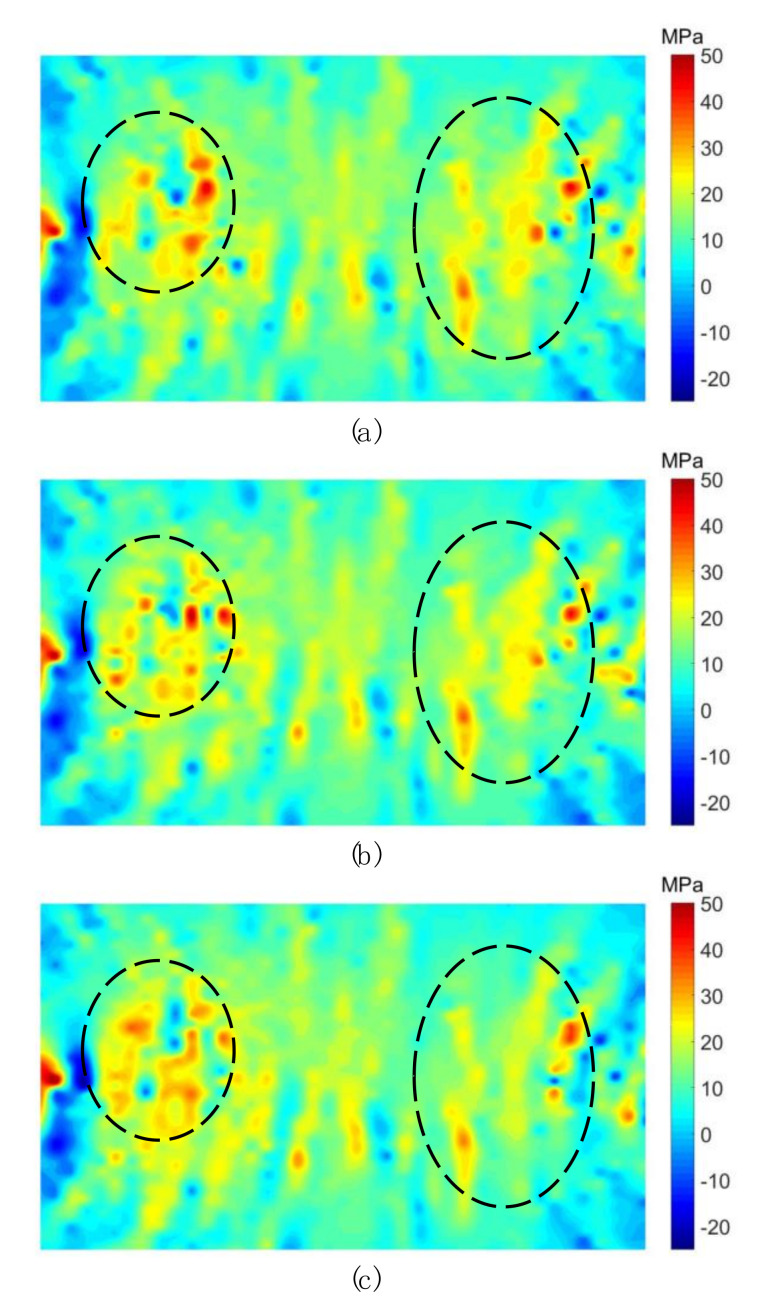
Distributions of the minor principal stress inside samples (I = 5) considering the loading rate of (**a**) 484 GPa/s; (**b**) 639 GPa/s; (**c**) 768 GPa/s. The positive presents the tensile stress and the negative denotes the compressive stress. For interpretation of the references to color in this figure, the reader is referred to the electronic version of this paper.

Figure 9 shows distributions of the minor principal stress of ZOI inside the sample (I = 5) considering different loading rates when the displacement between two loading points is 0.1 mm. For better comparison, we enclosed typical zones using dashed ellipses. As the loading rate increases, the TSC gradually fades away. That is, the magnitude of the TSC has a decreasing trend with increasing the loading rate and there is a transition from the sharp TSC to the smooth tensile stress distribution.

**Figure 10:**
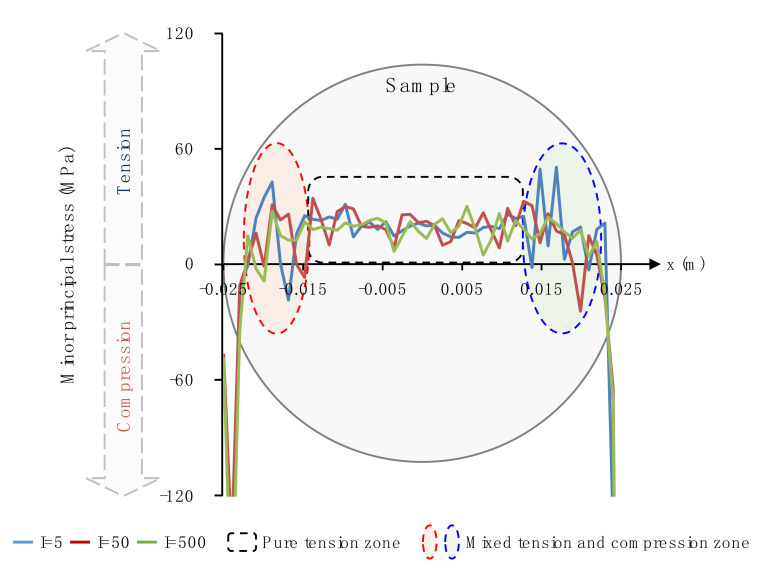
Distributions of the minor principal stress along the horizontal symmetrical line of samples considering different grain heterogeneity. The displacement between two loading points is 0.1 mm. For interpretation of the references to color in this figure, the reader is referred to the electronic version of this paper.

Figure 10 shows the minor principal stress along the horizontal symmetrical lines of samples considering different grain heterogeneity when the loading rate is around 390 GPa/s and the displacement between two loading points is 0.1 mm. It can be seen that areas adjacent to two loading points mainly suffer from the compressive stress with large magnitudes. Due to the grain heterogeneity, each minor principal stress curve presents fluctuations. It can be observed that the tensile stress only appears in the zone where x ranges from around −14.5 to 14 mm, named as the pure tension zone. In this zone, the fluctuation is relatively small and its amplitude only changes a little as the grain heterogeneity weakens. However, there exists two zones mixed with the tensile and compressive stress, named as the mixed tension and compression zone, which is different from the case of the homogenous sample [36], i.e., a smooth curve parallel with the symmetrical line. One is located in the zone where x ranges from around −22.5 to −14.5 mm; the other is located in the zone where x ranges from around 14 to 22.5 mm. In these two mixed zones, the fluctuation is far fiercer than that in the pure tension zone. With the grain heterogeneity weakened, the fluctuation amplitude has a decreasing trend, especially when I ranges from 5 to 50. It can be inferred that the grain heterogeneity mainly influences the transition zones from the compression to the tension, while it has a relatively limited influence on both the compression zone and the pure tension zone.

**Figure 11:**
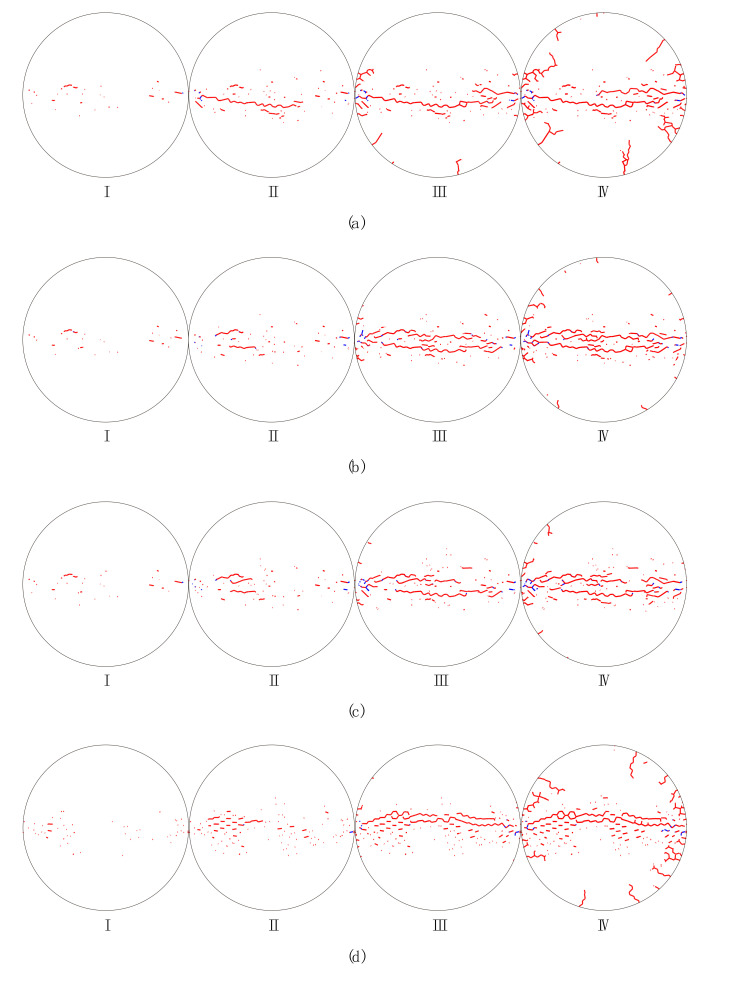

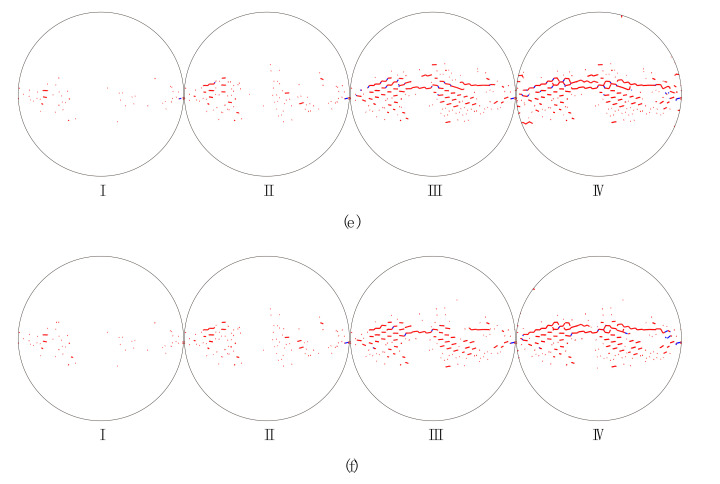
Progressive failure process of the sample considering different loading rates of (**a**) 394 GPa/s (I = 5); (**b**) 565 GPa/s (I = 5); (**c**) 704 GPa/s (I = 5); (**d**) 391 GPa/s (I = 500); (**e**) 579 GPa/s (I = 500); (**f**) 741 GPa/s (I = 500). Stages I, II, III, and IV correspond to the displacement between two loading points 1, 1.25, 1.5, and 1.75 mm. The red and blue lines denote the tensile and shear micro cracks with respect. For interpretation of the references to color in this figure, the reader is referred to the electronic version of this paper.

Figure 11 shows progressive failure processes of the synthetic crystalline rock sample subjected to different loading rates. In general, the cracking process seems to start from the left and right sides of the sample, and moves inward. In the first stage, tensile micro cracks essentially equally appeared along the central area of the sample. Lots of tensile micro cracks were initiatively coalesced into macro cracks and a few shear micro cracks began to emerge near two loading points in the second stage. In the third stage, micro tensile cracks were further coalesced into persistent macro cracks, while the propagation of the shear micro cracks was essentially prohibited. Finally, the sample was split into two halves in the fourth stage. The loading rate has an obvious influence on the progressive failure characteristics of the sample. When the loading rate is 394 GPa/s (Figure 11a), several tensile micro cracks emerge symmetrically on two margins slightly away from the left loading point in the third stage. Yet there only exists less of these tensile micro cracks as the loading rates are 565 GPa/s (Figure 11b) and 704 GPa/s (Figure 11c). In the fourth stage, there are two crushing zones adjacent to loading points for the case of a loading rate of 394 GPa/s (Figure 11a). With the loading rate increasing, these two crushing zones gradually disappear (Figure 11b,c). For the case of I = 500 (Figure 11d–f), the progressive failure characteristic is similar to that of I = 5, except that it has a wider failure band.

## 4. Discussion

In this study, we investigated the compression-induced tensile mechanical behaviors of the crystalline rock, combining the SHPB test and the GBM simulation. The experimental results showed that the macro tensile strength of the SJK granite depended on the loading rate, which was similar to findings obtained in previous studies [25,26,27]. However, previous studies rarely characterized the dynamic compression-induced tensile mechanical behaviors of the crystalline rock from the micromechanical viewpoint. It is thus not enough to understand the real physics of the crystalline rock. Our GBM model can make up for this limitation and micromechanically reveal the response of the crystalline rock to the dynamic compression-induced tension.

Via the GBM, we can consider effects of the grain heterogeneity and the loading rate on the mechanical behaviors of the crystalline rock under the dynamic compression-induced tension from the grain scale. Previous studies showed that the heterogeneity of the grain morphology significantly affected the stress distribution and progressive failure characteristics inside the synthetic granite under the static uniaxial compression [24]. In their study, TSCs and micro cracks essentially distributed equally inside the whole rectangle sample regardless of the variation of the grain heterogeneity. Nevertheless, distributions of TSCs and micro cracks inside the sample of the Brazilian tension test depended on both the grain heterogeneity and the loading rate from our current study. The ZOI in Figure 8 mainly suffered from the tensile stress, especially in subzone 2#. As the grain heterogeneity weakened, the number of TSCs in subzone 2# had a prominent increase, while it only had a limited variation in other two subzones. This indicates that subzone 2# is more heterogeneity-dependent for the tensile stress distribution compared with the other two subzones. Along the whole horizontal symmetrical line, the fluctuation in the mixed tension and compression zone was much fiercer than the pure tension zone (Figure 10). Hence, the mixed tension and compression zone actually has the most prominent effect of the grain heterogeneity on the compression-induced tensile behaviors. For the case of I = 5, the curve had the maximum tensile stress, while it had the minimum tensile strength for the case of I = 500 (Figure 10). We can now understand that the Brazilian tensile strength increased with the grain heterogeneity weakened (Figure 7). The transition from the sharp TSC to the smooth tensile stress distribution zone with increasing the loading rate could promote the increase in the Brazilian tensile strength and the microcracking along the narrow central line in an equal manner (Figure 9 and Figure 11). This may be one reason in which the sample had the rate-dependent Brazilian tensile strength (Figure 7). From the above, it can be concluded that the crystalline rock had the rate- and heterogeneity-dependency of compression-induced tensile mechanical behaviors. The heterogeneity-dependent characteristic was obviously enhanced as the loading rate increased, while the rate-dependent characteristic became strong with the grain heterogeneity weakened (Figure 7). These two factors affected the dynamic compression-induced tensile mechanical behaviors in a coupling mode.

The phenomenon in which the heterogeneity-dependency of the dynamic Brazilian tensile strength enhanced with increasing the loading rate is meaningful for the practical engineering such as underground protection works from blasting and strong seismic stress waves. Based on the grain heterogeneity characteristic of the surrounding crystalline rock, one can evaluate the dynamic tensile strength under different loading rates and thus, conduct the optimal design of protective structures.

In the current study, polygonal grains inside the synthetic crystalline rock sample were treated as small 2D blocks with the linearly elastic deformation behavior, which cannot consider the rate-dependent mechanical characteristics of minerals. On the other hand, intragranular and transgranular cracking inside the mineral grains of the crystalline rock were not taken into account. Furthermore, all mineral grain contacts were bonded interfaces, while some grain boundaries in the real weathered crystalline rock lost the bond strength and formed cracks. Thus, it is also necessary to investigate the influences of inside pre-existing cracks on macro- and micromechanical behaviors of the crystalline rock. Finally, 3D models also need to be considered. Therefore, further studies should be conducted to overcome above limitations.

## 5. Conclusions

Understanding the compression-induced tensile mechanical behaviors of the crystalline rock under dynamic loads is of great significance for the practical engineering such as drilling and blasting excavation. However, thus far, related studies have rarely been conducted to reveal these dynamic tensile characteristics of the crystalline rock from the micromechanical viewpoint. In this paper, we first conducted SHPB Brazilian tests to investigate the macro tensile mechanical behaviors of the SJK granite under dynamic loads. Then, the GBM by UDEC was established to micromechanically explore the compression-induced tensile mechanical response of the synthetic sample. Some important and interesting conclusions were addressed as follows.

The crystalline rock had the rate- and heterogeneity-dependency of the compression-induced tensile mechanical behaviors. Brazilian tensile strength increased as the grain heterogeneity weakened or the loading rate increased. The heterogeneity-dependent characteristic was obviously enhanced as the loading rate increased, while the rate-dependent characteristic became strong with the grain heterogeneity weakened. Due to heterogeneity, tensile stress concentrations (TSCs) existed inside the synthetic crystalline rock, especially in the mixed compression and tension zone, which could favor microcracking and strength weakening of the sample. As the grain heterogeneity weakened or the loading rate increased, the magnitude of the TSC had a decreasing trend and there was a transition from the sharp TSC to the smooth tensile stress distribution zone. The progressive failure of the crystalline rock was notably influenced by the loading rate, which mainly represented the formation of the crushing zone adjacent to the two loading points. Our results are meaningful for the practical engineering, such as underground protection works from stress waves.

## Figures and Tables

**Table 1:** Comparisons of mineral compositions between the Shuangjiangkou (SJK) granite sample and the corresponding synthetic sample by the grain-based model (GBM).

Mineral Type	Actual (%)	Synthetic (%)	Error (%)
K-feldspar	40.0	40.1	+0.25
Quartz	25.0	24.9	−0.40
Plagioclase	30.0	30.0	0.0
Biotite	5.0	5.0	0.0

**Table 2:** Physical and mechanical properties of different minerals in the synthetic granite (from reference [34]) and the steel bar.

Mineral Type (Bar)	Density (g/cm^3^)	Elastic Modulus *E* and (*K*, *G*) ^1^ (GPa)	Poisson’s Ratio
K-feldspar	2.56	69.8 (53.7, 27.2)	0.28
Quartz	2.65	94.5 (37, 44)	0.08
Plagioclase	2.63	88.1 (50.8, 29.3)	0.26
Biotite	3.05	33.8 (41.1, 12.4)	0.36
Steel bar	7.90	184.4 (140, 72)	0.28

^1^*K*—bulk modulus; *G*—shear modulus.

**Table 3:** Calibrated micro contact parameters of the numerical model.

Parameters	Grain Contact	Sample-Bar Contact
*k*_n_ (Pa/m)	5.0 × 10^14^	5.0 × 10^14^
*k*_s_ (Pa/m)	2.5 × 10^14^	2.5 × 10^14^
*T* (MPa)	43.0	0
*c* (MPa)	120.0	0
*φ* (°)	27.0	0
